# Novel Qnr Families as Conserved and Intrinsic Quinolone Resistance Determinants in *Aeromonas* spp.

**DOI:** 10.4014/jmb.2403.03043

**Published:** 2024-04-19

**Authors:** Sang-Gyu Kim, Bo-Eun Kim, Jung Hun Lee, Dae-Wi Kim

**Affiliations:** 1 Department of Life Sciences, Jeonbuk National University, Jeonju 54896, Republic of Korea; 2 Microbial Safety Division, National Institute of Agricultural Sciences, Rural Development Administration, Wanju 55365, Republic of Korea; 3 National Leading Research Laboratory of Drug Resistance Proteomics, Department of Biological Sciences, Myongji University, Yongin 17058, Republic of Korea

**Keywords:** *Aeromonas* spp., quinolone resistance, Qnr protein, comparative genomics

## Abstract

The environment has been identified as an origin, reservoir, and transmission route of antibiotic resistance genes (ARGs). Among diverse environments, freshwater environments have been recognized as pivotal in the transmission of ARGs between opportunistic pathogens and autochthonous bacteria such as *Aeromonas* spp. In this study, five environmental strains of *Aeromonas* spp. exhibiting multidrug resistance (MDR) were selected for whole-genome sequencing to ascertain their taxonomic assignment at the species-level and to delineate their ARG repertoires. Analyses of their genomes revealed the presence of one protein almost identical to AhQnr (*A. hydrophila* Qnr protein) and four novel proteins similar to AhQnr. To scrutinize the classification and taxonomic distribution of these proteins, all *Aeromonas* genomes deposited in the NCBI RefSeq genome database (1,222 genomes) were investigated. This revealed that these *Aeromonas* Qnr (AQnr) proteins are conserved intrinsic resistance determinants of the genus, exhibiting species-specific diversity. Additionally, structure prediction and analysis of contribution to quinolone resistance by AQnr proteins of the isolates, confirmed their functionality as quinolone resistance determinants. Given the origin of mobile *qnr* genes from aquatic bacteria and the crucial role of *Aeromonas* spp. in ARG dissemination in aquatic environments, a thorough understanding and strict surveillance of AQnr families prior to the clinical emergence are imperative. In this study, using comparative genome analyses and functional characterization of AQnr proteins in the genus *Aeromonas*, novel *Aeromonas* ARGs requiring surveillance has suggested.

## Introduction

Since the discovery of antibiotics and their introduction to clinical settings, infectious diseases caused by bacteria have been perceived to be under control [1]. However, the efficacy of these drugs has been threatened by the continuous emergence of antibiotic resistance genes (ARGs) and their rapid dissemination, highlighting the antibiotic resistance crisis [2]. The concept of the antibiotic resistome, first proposed in 2006 [3], has led to significant advancements in the field of environmental antibiotic resistome, underscoring the significance of environment as origin and dissemination route of ARGs [4]. Moreover, antibiotic resistance in the environment must be diverse, complex and ever expanding [5, 6]. Recent studies emphasized the importance of characterizing novel ARGs in environmental bacteria prior to their emergence in clinical settings, facilitating the identification of potential threats for surveillance purposes [6, 7].

*Aeromonas*, Gram-negative, facultative anaerobic, and rod-shaped bacterium belonging to the family Aeromonadaceae, encompasses 32 valid species within the genus. *Aeromonas* spp. are ubiquitous and autochthonous to diverse aquatic environments, including freshwater, estuaries, surface water, and sewage [8]. While the genus *Aeromonas* has been primarily recognized as fish pathogens [9], certain studies have reported *Aeromonas* spp. as human opportunistic pathogens [10, 11]. Among *Aeromonas* species, *A. hydrophila* is the most prominent pathogen, implicated not only in gastroenteritis and skin infection, but also in more systemic conditions such as bacteremia and endocarditis [12-14]. Additionally, *A. caviae*, *A. veronii*, and *A. dhakensis* are recognized as potential human pathogens [15, 16]. Importantly, the genus *Aeromonas* serves as a reservoir and important dissemination route for various ARGs in aquatic environments [17, 18]. Indeed, a study on the resistome of water system have highlighted the pivotal role of *Aeromonas* spp. in proliferation and dissemination of ARGs in aquatic ecosystems [19]. From the perspective of One Health surveillance, aquatic environments are one of the most critical habitats and transmission routes for antibiotic-resistant bacteria (ARB) and ARGs, contributing to the dissemination of antibiotic resistance across the One Health sectors (human, animal, and environment) [6, 20, 21]. Quinolone antibiotics, fully synthetic drugs, worked as inhibitors of bacterial replication, have found extensive applications in clinical and veterinary medicines [22, 23]. Operating by interacting with DNA gyrase and topoisomerase IV, they disrupt bacterial DNA replication, induce damage DNA and cell death [24]. They have been used to treat various bacterial infections caused by both Gram-positive and -negative bacteria [25]. The increasing usage of fluoroquinolones has led to the emergence and dissemination of ARGs against the drugs in the clinical or veterinary settings [24, 26]. In particular, quinolone resistances genes such as genes encoding Qnr family proteins, AAC(6’)-Ib-cr, and efflux pumps (QepA and OqxAB), have been defined as plasmid-mediated quinolone resistance (PMQR) determinants [27], frequently encountered in plasmids of diverse clinical isolates. Among these PMQR proteins, Qnr family proteins are categorized into seven families as follows: QnrA, QnrB, QnrC, QnrD, QnrE, QnrS, and QnrVC [28]. The presence of *qnr* gene in a plasmid as PMQR was first identified in the transferable plasmid of β-lactam-resistant clinical isolates of *K. pneumoniae* and *E. coli* [29]. Qnr proteins confer resistance to fluoroquinolone by wrapping around the antibiotic targets (DNA gyrase and topoisomerase IV), thereby impeding drug access [30]. Apart from PMQR Qnr families, Qnr-like proteins featuring pentapeptide repeats are indigenous to the chromosomes of diverse Gram-positive and -negative bacteria. Examples include MfpA of *M. tubeculosis*, EfsQnr of *Enterococcus faecalis*, SmQnr of *Stenotrophomonas maltophilia*, SmaQnr of *Serratia marcescens*, and AhQnr of *Aeromonas hydrophila* [31-36]. Moreover, PMQR Qnr proteins have been considered an antibiotic resistance originating from aquatic environmental bacteria due to reports detailing the intrinsic existence of *qnrA* genes in *Shewanella algae* as a progenitor [37]. In the genus *Aeromonas*, several reports have documented the presence of Qnr proteins as both PMQR and chromosome-borne determinants, exemplified by QnrS and QnrVC of *Aeromonas* spp., and AhQnr of *A. hydrophila* [36, 38, 39]. Protein sequences of chromosome-borne Qnr proteins of *Aeromonas* were shown to be distinguished with both those of PMQR Qnr proteins and chromosome-borne Qnr proteins of other genera [31, 40]. Nevertheless, a comprehensive understanding of classification and taxonomic distribution of these *Aeromonas*-originated Qnr proteins remains lacking.

In this study, using complete genome sequences of five *Aeromonas* isolates from various environments, Qnr-like proteins were identified and compared with Qnr-like proteins of all *Aeromonas* genomes deposited in the NCBI RefSeq database to provide sequence-based classification and taxonomic distribution of Qnr proteins in *Aeromonas* species. Moreover, among those, novel Qnr candidates were elucidated and they were functionally characterized using structure prediction and quinolone resistance analyses, suggesting novel intrinsic Qnr proteins of the genus *Aeromonas*.

## Materials and Methods

### Sampling

Environmental sediment samples were collected from three regions of South Korea to isolate environmental ARB in December 2019. Detailed information regarding sampling sites and sample types is provided in Table S1. All samples were collected in sterilized container and transferred to the laboratory within 8 h.

### Isolation and Identification of Environmental Bacteria

A suspension of 1 g of samples was prepared in 9 ml of R2A broth media (MBcell, Republic of Korea) and it was serially diluted 10-fold. Subsequently, 100 μl of diluted samples were spread onto CHROMagar ECC and CHROMagar *Pseudomonas* media (CHROMagar, France) for isolate environmental ARBs. The plates were then incubated at 30°C for 24 h. Distinct colonies were selected based on the morphological characteristics. Pure cultures obtained after several subcultures of selected colonies were preserved at -80°C as glycerol stocks (30%glycerol). Genomic DNA extraction from the samples was conducted using DNeasy Blood & Tissue kit (Qiagen, USA) according to the manufacturer’s instruction. The 16S rRNA gene of the extracted DNA was amplified by polymerase chain reaction (PCR) using universal primers 27F and 1492R [41]. The amplicons of 16S rRNA were sent to BioFact Co. (Republic of Korea) for sequencing. The obtained 16S rRNA gene sequences were submitted to EzBioCloud server (https://www.ezbiocloud.net) to identify the strains at the genus-level [42]. Strains identified as belonging to the genus *Aeromonas* were selected for further study.

### Antibiotic Susceptibility Test Using Disk-Diffusion Assay

A total of 18 antibiotics were employed for antimicrobial susceptibility test (AST) of *Aeromonas* spp. to evaluate their antibiotic resistance using the disk-diffusion assay according to Clinical & Laboratory Standards Institute (CLSI) guideline (Table S2) [43]. Environmental *Aeromonas* strains were cultured overnight at 30°C in Muller-Hinton broth (MHB, BD Difco, USA). The overnight culture was then transferred to fresh MHB medium and incubated at 37°C until the optical density (OD) reached to 0.1 at 625 nm. Subsequently, the culture was spread on Muller-Hinton agar (MHA, BD Difco) plate using sterile cotton swab. After the plates had dried, antibiotic disks were placed on the agar plate. The plates were incubated at 30°C for 20 h. Antibiotic susceptibility was determined based on the size of the inhibition zone where bacterial growth was absent. *Aeromonas* spp. exhibiting a multidrug resistance (MDR) phenotype were selected for genome sequencing.

### Whole Genome Sequencing, Assembly and Annotation

Genomic DNAs of selected MDR *Aeromonas* spp. were extracted following the previously described method. The quality of the extracted DNA was evaluated through agarose gel electrophoresis and nanodrop analyses (Biotek, USA). For whole genome sequencing *via* single-molecule real-time (SMRT) platform (PacBio RSII, Pacific Science, USA), DNA quality was further assessed in accordance with the sequencing company's guides (DNA Link, Republic of Korea). Samples meeting the quality control standards were used to construct library using SMRTbell library prep kit 1.0 (Pacific Science). Additionally, the NovaSeq 6000 system (Illumina, USA) was employed to correct errors inherent in the SMRT platform and to obtain complete genome sequences. Whole genome sequence of bacteria was generated using the sequencers, and *de novo* genome assembly was conducted with HGAP3 software (v2.3) [44]. The completeness of the genome assembly data was evaluated using Benchmarking Universal Single-Copy Orthologs (BUSCO). The complete genome sequences of five environmental *Aeromonas* spp. were annotated using Prokka annotation tool [45]. Complete genome sequences of *Aeromonas* spp. obtained in this study have been deposited in the GenBank under accession numbers of CP149130 (chromosome of strain BE1), CP149124 (chromosome of strain BE2), CP149125 (chromosome of strain BE3), CP149126 (plasmid of strain BE3), CP149127 (chromosome of strain BE4), CP149128 (plasmid of strain BE4), and CP149129 (chromosome of strain BE4).

### Genome-Based Phylogeny of *Aeromonas* spp.

Average nucleotide identity (ANI) was calculated using the FastANI tool [46]. For the precise taxonomic assignment of five isolates at the species-level through phylogenetic tree construction, genome sequences of reference strains of 32 *Aeromonas* species, were used in conjunction with those of the five strains. For the construction of a phylogenomic tree encompassing all *Aeromonas* genomes, a total of 1,222 genomes deposited in the NCBI RefSeq database (as of Nov. 2023) were used. Core genes, present in more than 99% of the genomes, were extracted from the pan-genome of genome sets for each analysis using the PIRATE toolbox [47]. These core genes were then aligned and concatenated using the MAFFT program [48]. Using the aligned sequences, the tree was constructed by the FastTree 2 [49] and visualized by iTOL [50].

### Resistome Analysis of Isolated *Aeromonas* spp.

The analysis of ARG contents in the genomes of environmental *Aeromonas* spp. was conducted using the resistance gene identifier (RGI) integrated within the Comprehensive Antibiotic Resistance Database (RGI v6.0.3, CARD v3.2.9.) [51]. Only a homology model was employed to identify ARGs in the genomes. For the detection of previous characterized ARGs, strict criteria were applied in RGI analyses, whereas for the identification of novel ARG candidates, hits representing a ratio of the best bit score to the pass bit score greater than 0.4, were considered potential ARGs (relaxed criteria).

### Qnr Classification and Phylogeny

Qnr proteins were systematically searched in all *Aeromonas* genomes in the database using the RGI, employing relaxed criteria as previously described. Qnr proteins from both *Aeromonas* genomes and the CARD, were subjected to clustering using CD-HIT with 95% cutoff [52]. The resulting clusters specific to *Aeromonas*, denoted as AQnr, were assigned numerical identifiers based on their abundance rank. Representative sequences from each cluster were selected, and a phylogenetic tree of these Qnr proteins was constructed using the MAFFT and FastTree 2 [47, 48]. Information regarding Qnr classification was then incorporated into the phylogenomic tree of all *Aeromonas* genomes using iTOL [49].

### Structural Prediction of Novel Qnr Proteins

The structures of representative sequences of AQnr families were predicted using the ColabFold platform [53]. The predicted structures of AQnr proteins were compared with those of previously characterized Qnr proteins (AhQnr (PDB: 3PSS), QnrB1 (PDB: 2XTW), MfpA (PDB: 6ZT4)) obtained from the protein databank (PDB). This comparison was visualized and analyzed using the UCSF ChimeraX software [54]. In addition, a structure-based alignment of Qnr proteins was performed to assess the conserveness of pentapeptide repeats, faces, coils, and loops, following analytical criteria in previous studies [55, 56].

### Analyses of Quinolone Rsistance Conferred by AQnr Proteins

The genes encoding AQnr proteins were amplified by PCR from chromosomal DNA of environmental *Aeromonas* spp. using appropriately designed primers (Table S3). The amplified DNAs and the intact pHSG398 plasmid were cut by appropriate restriction enzymes and purified using QIAquick PCR & Gel Cleanup Kit (Qiagen) (Table S3). The purified PCR product and the linearized plasmid were ligated using T4 DNA ligase (New England BioLabs, USA) and the reaction mixture was transformed into *E. coli* TOP10 using a heat shock method [57]. The transformants were selected on Luria-Bertani (LB) agar media containing a chloramphenicol antibiotic (34 μg/ml). From the transformants, plasmid DNA was extracted, and the presence of target DNA in the plasmid was verified by cutting with appropriate restriction enzymes (Table S3). The verified plasmid DNAs were sent to the sequencing company (BioFact, Republic of Korea) to confirm the sequences. The contribution of Qnr proteins to quinolone resistance was evaluated by analyzing changes in the resistance of *E. coli* carrying *qnr* genes compared to control strains (*E. coli* TOP10 and *E. coli* TOP10 carrying intact pHSG398 plasmid). The agar dilution method was employed to determine minimum inhibitory concentrations (MIC) as described previously [43]. Briefly, overnight cultured strains in MHB were transferred into fresh MHB with a ratio of 1% inoculum size. After incubation at 37°C on a shaker at 225 rpm up to an OD at 625 nm of 0.1 (McFarland 0.5), the culture was diluted 10-fold and applied as 1 μl drops on the surface of MHA plates containing different concentrations of quinolone antibiotics. Eight quinolone antibiotics were used for AST: nalidixic acid, norfloxacin, ofloxacin, ciprofloxacin, levofloxacin, moxifloxacin, enrofloxacin, and sarafloxacin.

## Results

### Isolation and Identification of MDR *Aeromonas* spp. from the Environment

**Fig. 1:**
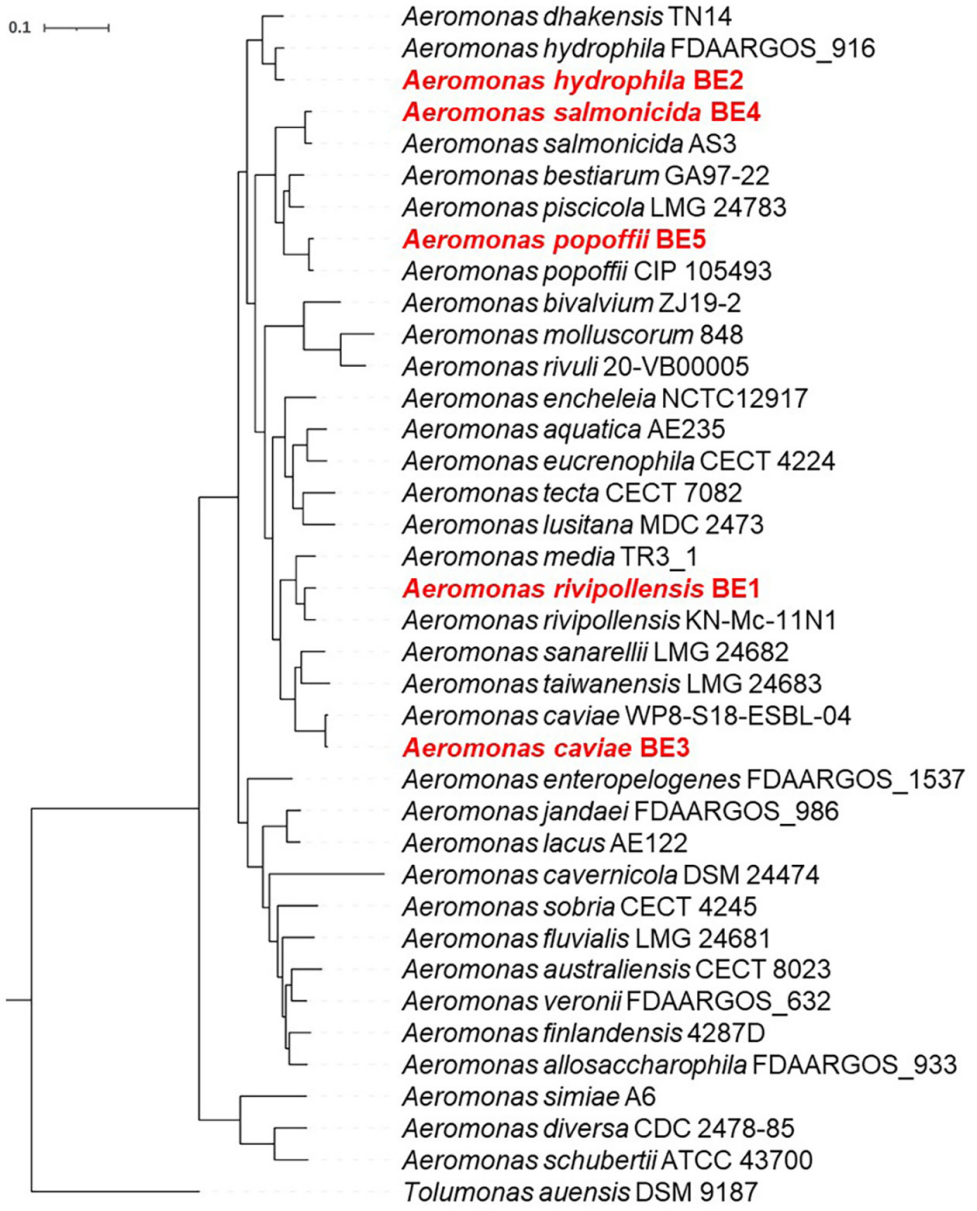
Phylogenomic tree of *Aeromonas* spp. isolates. The tree was constructed using the maximum-likelihood method, employing a set of 996 core genes derived from genomes of 32 reference strains of the genus Aeromoans and genomes of the five isolates. *Tolumonas auensis* DSM 9187 was used as an outgroup. The taxonomic names of the isolates were indicated in red.

A total of 424 bacterial isolates from environmental sites were identified at the genus-level using their 16S rRNA gene sequences through the EzBioCloud database (Table S1). Among these, 35 strains were identified as belonging to the genus *Aeromonas*. Antibiotic resistance profiles, employing 18 commonly used antibiotics, revealed a subset of five strains exhibiting MDR phenotype (Fig. S1). Designated as strains BE1, BE2, BE3, BE4, and BE5, these MDR *Aeromonas* strains were subjected to whole genome sequencing, and their complete genome information was shown in Table S4. Strains BE3 and BE4 were found to carry an additional plasmid DNA, as 53,669 and 37,925 bp length, respectively (Table S4). The 16S rRNA sequences of strains BE1, BE2, BE3, BE4, and BE5 exhibited the highest similarity to type strains *A. media* CECT 4232^T^ (99.86%), *A. hydrophila* ATCC 7966^T^ (99.80%), *A. caviae* CETC 838^T^ (99.93%), *A. salmonicida* ATCC 33658^T^ (100.00%), and *A. popoffii* CIP 105793^T^ (100.00%), respectively. The ANI values between the genomes of MDR *Aeromonas* strains and those of reference strains were higher than the species delineation threshold (95~96%) (Table S5) [46, 58]. In the case of strain BE1, although an initial assignment based on the 16S rRNA gene suggested to be belonging to A.media, the ANI value between strain BE1 and A.media was 93.81%, lower than that between strain BE1 and *A. rivipollensis* (96.19%). Furthermore, strain BE1 exhibited a closer clustering with *A. rivipollensis* than *A. media* in the genome tree (Fig. 1). The identity of 16S rRNA gene sequence between strain BE1 and *A. rivipollensis* was also exceeded the species delineation threshold (99.34%). To ensure accurate taxonomic assignment, a phylogenomic tree was constructed using a set of 996 core genes from 32 reference genomes encompassing all species within the genus *Aeromonas* (Fig. 1). Consequently, the five environmental *Aeromonas* strains were taxonomically assigned as *A. rivipollesis* strain BE1, *A. hydrophila* BE2, *A. caviae* BE3, *A. salmonicida* BE4, and *A. popoffii* BE5.

### Resistome Analyses Based on Genome Sequences of MDR *Aeromonas* spp.

To elucidate the MDR phenotypes of these *Aeromonas* spp., an analysis of their ARG repertoires based on genome sequences was conducted (Table 1). Strains were found to carry ARGs ranging 4-7 in their genomes when strict criteria in the RGI analysis was applied. According to target antibiotic classification, genes encoding b-lactamases as b-lactam resistance genes, were identified across all strains and *bla*_TRU-1_ gene was conserved in strains BE1, BE4, and BE5. A peptide antibiotic resistance gene (*arnT*) was present in strains BE2 and BE4. Strain BE3 uniquely harbored a tetracycline resistance gene (*tet(E)*) and a phosphonic acid antibiotic resistance gene (*fosA8*). A glycopeptide resistance gene (*vanT*) and multidrug resistance efflux pump genes (*adeF* and *rsmA*) were detected in all strains of various species, indicating their conservation as intrinsic ARGs in the genus *Aeromonas* (Table 1). However, no quinolone resistance gene was identified, and all detected ARGs were located in the chromosomes, not in plasmid, suggesting the absence of PMQR determinants in these isolates.

To identify novel ARG candidates, relaxed criteria were applied in the RGI analysis. Several potential ARGs were predicted and notably, genes encoding Qnr-like proteins were found in all strains. Designated as AQnrBE1, AQnrBE2, AQnrBE3, AQnrBE4, and AQnrBE5 to denote their *Aeromonas*-origin and respective strain designation. As they were not detected in the first RGI search (with strict criteria), they exhibited low amino acid sequence identity (less than 50%) to know Qnr family proteins (QnrA, QnrB, QnrC, QnrD, QnrS, QnrVC, QnrE, and mfpA) deposited in the CARD (AhQnr is absent in the CARD). When comparing to AhQnr, AQnrBE2 displayed nearly identical sequence to AhQnr (99.07%), whereas the others showed identities ranging from 83.80 to 86.11%to AhQnr, suggesting their potential novelty, which is related to AhQnr rather than the other Qnr proteins in the database. Furthermore, sequence identities among AQnrBE1, AQnrBE3, AQnrBE4, and AQnrBE5 ranged from 83.33% to 92.59%, indicating their sequence variations corresponding to their species origin.

### Novel Qnr Family Proteins Conserved in *Aeromonas* spp.

Numerous studies have highlighted the presence of QnrS and QnrVC proteins as PMQR determinants *Aeromonas* spp. [38, 39]. However, the protein sequences of AQnr proteins including AhQnr, were markedly differ from previously characterized Qnr families in the CARD. With the exception of AhQnr, the functionality of these AQnr proteins remains unexplored [36]. While genes encoding Qnr-like pentapeptide repeat proteins are frequently annotated in the genomes of *Aeromonas* spp., their classification based on protein sequence and taxonomic distribution has not been defined, underscoring the necessity to scrutinize Qnr family proteins in *Aeromonas*.

To address the classification and distribution of Qnr proteins in *Aeromonas* spp., the presence of Qnr proteins in all *Aeromonas* spp. genomes deposited in the NCBI RefSeq database (as of Nov. 2023), was investigated. The majority of *Aeromonas* spp. genomes (98.0%, 1,198 out of 1,222 genomes) contain genes encoding Qnr-like proteins, which were categorized into two groups based on their protein sequence: Qnr proteins deposited in the CARD and AQnr proteins. In the case of CARD Qnr proteins, QnrS, QnrVC, QnrA, QnrB, and QnrD proteins were detected across various species, they were found in only 9.6% of genomes (117 out of 1,222 genomes). The presence of these highly similar proteins across different species suggests that they have been acquired ARGs in *Aeromonas* spp. through horizontal gene transfer, consistent with their prevalence in various genera [59]. In contrast, AQnr proteins (including AhQnr) were conserved in *Aeromonas* genomes, being present in 97.6% of the genomes (1,193 out of 1,222 genomes). Their sequence variations according to taxonomy suggested that AQnr proteins represent intrinsic resistance determinants of *Aeromonas* spp. Collectively, in the genus *Aeromonas*, two groups of Qnr proteins are present in their genome as acquired ARGs (Qnr proteins in the CARD) and intrinsic ARGs (AQnr proteins). To clarify this classification, all Qnr protein sequences from *Aeromonas* genomes and the CARD were collected and clustered using 95% identity cutoff, resulting in 46 Qnr protein clusters (Table S6). Among these, 32 Qnr proteins (including AhQnr) were not clustered with previously characterized Qnr proteins of the CARD, and were designated as AQnr families.

**Fig. 2:**
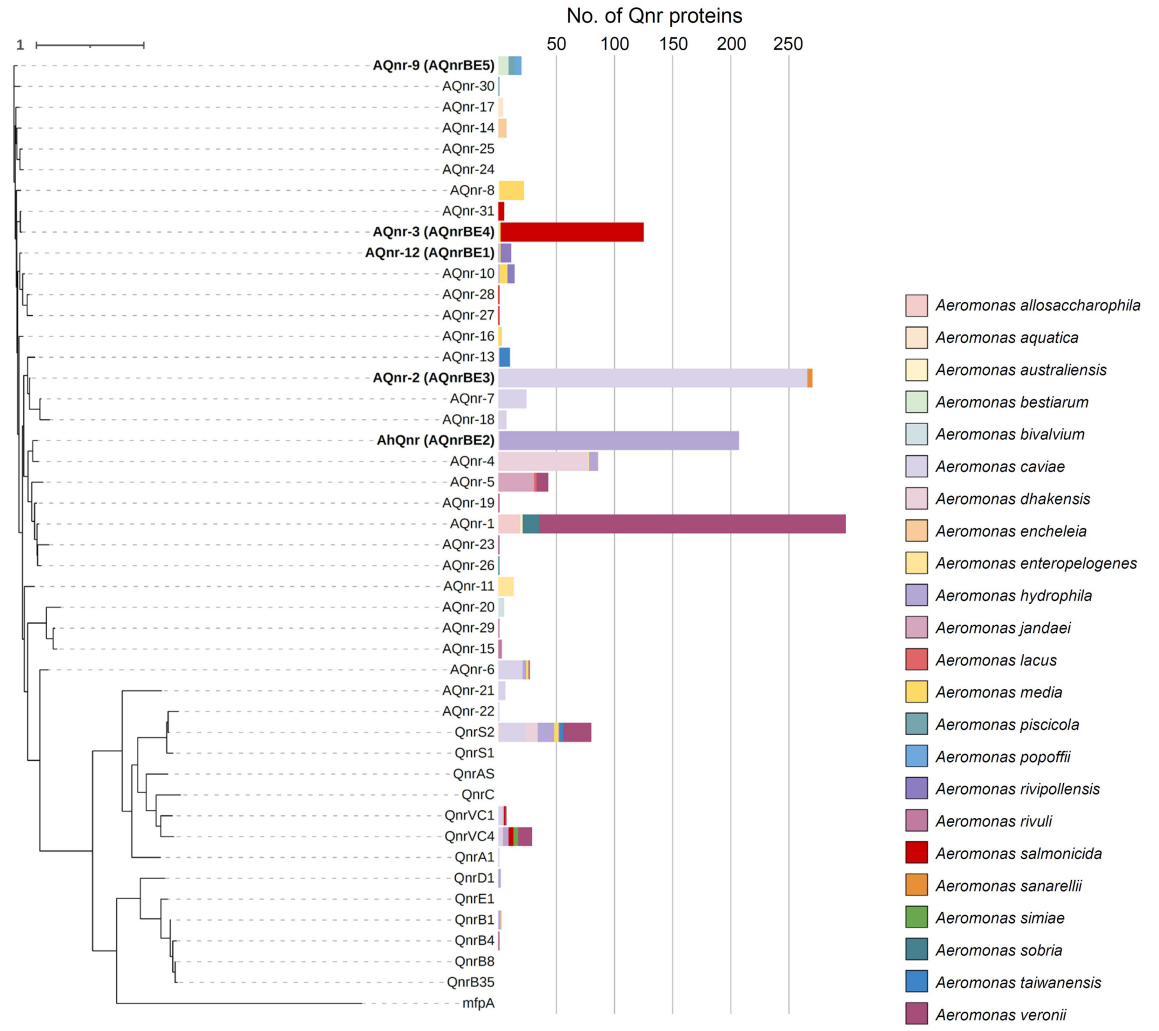
Phylogenetic tree of Qnr proteins and their distribution in *Aeromonas* speices. The phylogenetic tree of Qnr proteins was constructed using the maximum-likelihood method, employing all representative sequences of 46 clusters. These clusters were derived using 95% identity cutoff applied to *Aeromonas* Qnr proteins (AQnr) and Qnr proteins deposited in the comprehensive antibiotic resistance database. The taxonomic distribution of Qnr proteins in *Aeromonas* species was displayed by the number of cases of each species carrying each cluster. AQnr families containing AQnr proteins of the five isolates (AQnrBE1, 2, 3, 4, and 5) were indicated in bold.

Using the representative protein sequences of 46 clusters, the phylogenetic tree of Qnr proteins was constructed, clearly distinguishing between Qnr proteins from the CARD and AQnr families (Fig. 2). Regarding their taxonomic distribution, among acquired Qnr proteins, QnrS2, QnrVC4, and QnrVC1 were the most prevalent proteins across various species. In the case of other acquired Qnr proteins, QnrB1, QnrD1, QnrB4, and QnrA1, they are present only in the limited number of taxa (Fig. 2). However, their cases are rare (less than 3 cases for each), indicating their recent introduction to this genus compared to QnrS and QnrVC families. Notably, AQnr-21 and AQnr-22 families were solely identified in *A. hydrophila*, closely resembling acquired Qnr proteins rather than other AQnr families (identity between AQnr-21 and QnrVC1, 61%; identity between AQnr-22 and QnrS2, 89%), suggesting that these families represent variants of acquired Qnr proteins specific to *A. hydrophila*.

**Fig. 3:**
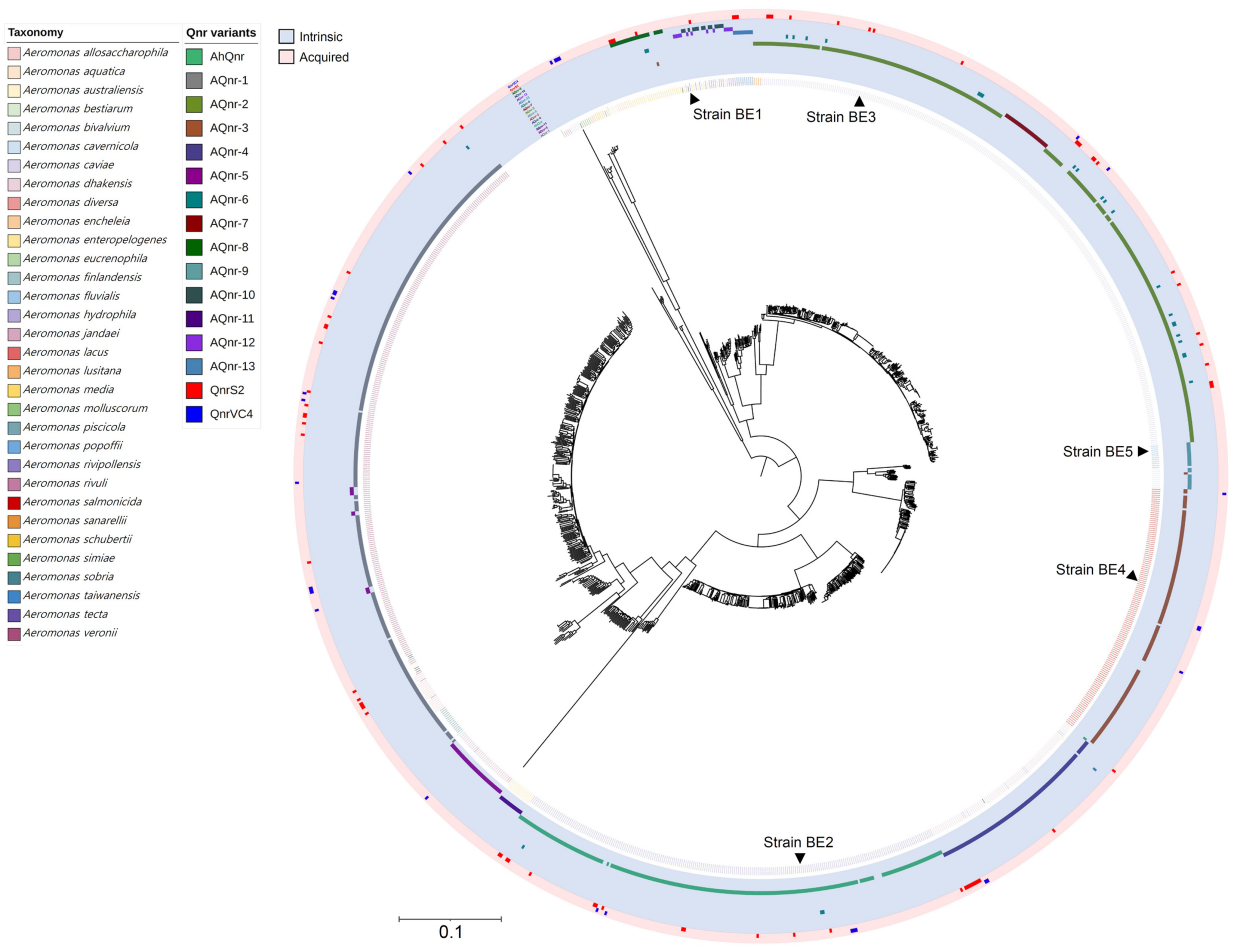
Distribution of AQnr proteins in *Aeromonas* spp. The phylogenomic tree was constructed using the maximum-likelihood method, based on a set of 217 core genes from genomes of 1,222 *Aeromonas* spp. from the NCBI RefSeq database and genomes of the five *Aeromonas* spp. isolates. The phylogenomic locations of the five isolates were marked with arrows and their strain names. Species-level taxonomy was indicated at strain names of the tree with colors. A total of 16 Qnr families covering 95% of all Qnr proteisn in *Aeromonas* spp., were shown at circle layers outside of the tree. Shades indicate the discrimination between acquired Qnr proteins (QnrS2 and QnrVC4) and intrinsic AQnr proteins.

In the case of AQnr families, they exhibited substantial diversity in terms of species-dependent family distribution. Some exceptions to this distribution were observed due to the indiscriminate clustering of AQnr families with 95% sequence identity in closely related species. For instance, AQnr-1, AQnr-6, and AQnr-9 were found in more than two species closely related in phylogenomic tree (Figs. 2 and 3). AQnr proteins identified in this study were categorized based on this family classification. AQnrBE2 was assigned to AhQnr family, while AQnrBE1, AQnrBE3, AQnrBE4, AQnrBE5 were clustered within AQnr-12, AQnr-2, AQnr-3, and AQnr-9 families, respectively. The species origins of these families coincided with the species of isolates (Fig. 2). These results endorsed that AQnr families are conserved and intrinsic in *Aeromonas* spp., evolving within the genus.

To assess the distribution of Qnr families among *Aeromonas* genomes, a phylogenomic tree of 1,227 *Aeromonas* genome, including the isolates from this study, was constructed based on 217 core genes. The Qnr protein repertoires of each genome were then mapped onto the tree. Among 46 Qnr clusters, which encompass both acquired Qnr proteins and intrinsic AQnr families, 16 major Qnr families covering over 95% of all Qnr proteins of *Aeromonas* spp. delineated in the tree (Fig. 3). As previously noted, intrinsic Qnr families (AQnr) exhibited species-specific distribution, whereas acquired Qnr families such as QnrS2 and QnrVC2 were found across diverse species (Fig. 3). Notably, in the case of *A. caviae*, two distinct types of AQnr families were identified. Strains within a specific phylogenetic branch of *A. caviae* were carrying the AQnr-7 instead of AQnr-2. The sequence identity of these two proteins is 90.3%, indicating their distinctiveness within the species (Fig. 3). More intriguingly, in *A. caviae*, 5.9% (21 out of 354 *A. caviae* genomes) of strains harbored an additional Qnr family (AQnr-6) as a paralog together with AQnr-2. AQnr-6 was also distributed in another 3 species (*A. hydrophila*, *A. media*, and *A. veronii*), which are phylogenetically distant from *A. caviae* (Figs. 2 and 3). AQnr-6 was not found in any other genera, except for a pentapeptide repeat-containing protein in *Acinetobacter* sp. genome (NCBI accession No. NZ_JAALGU010000190.1), which displayed a relatively a lower similarity (69.4%), than those of *Aeromonas* spp. These findings suggest that while AQnr families are currently intrinsic to *Aeromonas*, some are undergoing horizontal gene transfer within the genus *Aeromonas*. Additionally, we investigated the One Health origins of all *Aeromonas* spp. using the NCBI BioSample database to elucidate the origin and dissemination route of acquired Qnr proteins, such as QnrS2 and QnrVC4. However, it revealed no substantial correlation between isolation sources and these Qnr proteins, indicating that the prevalence of acquired Qnr proteins across the One Health sectors.

### Structural Characteristics of Novel Qnr Proteins

**Fig. 4:**
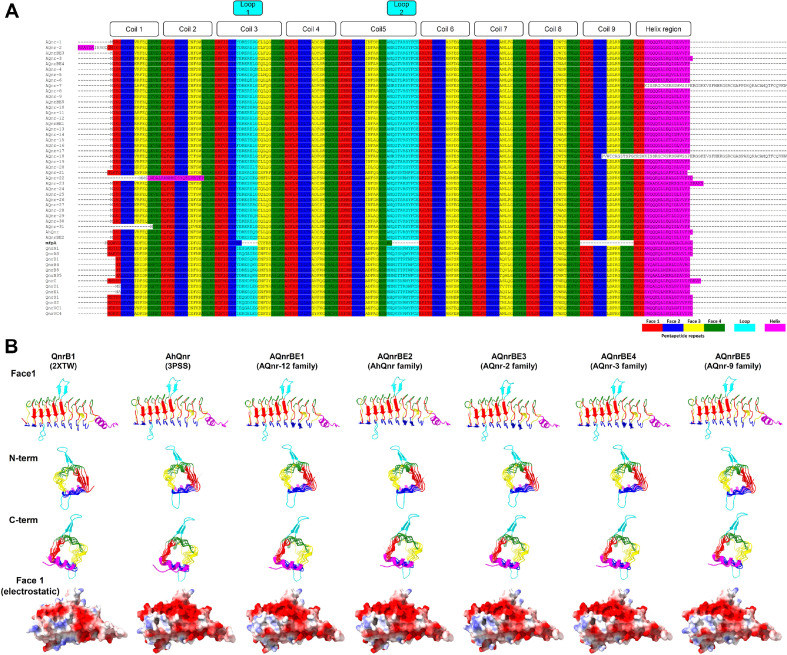
Structure based alignment and structure prediction of AQnr proteins of *Aeromonas* spp. isolates. (**A**) Structure based alignment of representative sequences of 32 AQnr families and 14 Qnr families from CARD. Each Qnr proteins consist of distinct three regions including coil, loop, and helix regions. The three regions and four faces of pentapeptide repeat were color-coded (face 1, red; face 2, blue; face 3, yellow; face 4, green; loop, cyan; helix, magenta). (**B**) Comparison of the overall structures of two Qnr proteins (QnrB1 and AhQnr) from the PDB and five AQnr proteins of the isolates. The structures of these AQnr proteins were predicted using the ColabFold platform, and depicted using UCSF ChimeraX software. The first to third panels show ribbon diagrams of each Qnr families looking at the different position (first, face 1; second, N-terminal; third, C-terminal). The last panel shows the electrostatic surface of each Qnr families.

The overall structures of AhQnr (PDB: 3PSS), QnrB1 (PDB: 2XTW), MfpA (PDB: 6ZT4), and EfsQnr (PDB: 2W7Z) proteins, along with their contribution to quinolone resistance, have been previously reported [32, 36, 60, 61]. Therefore, we aimed to predict the structures of AQnr proteins and to compare them with AhQnr and QnrBE1 to elucidate functional characteristics of these novel quinolone resistance determinants. Furthermore, since AQnr proteins from five isolates originated from phylogenetically diverse species, the structural and functional characterization of these proteins could offer a functional insight into the overall AQnr families (Figs. 2 and 3). Structures of all of representative proteins of 32 AQnr families were predicted using the ColabFold platform, and their structures were compared to those of AhQnr, QnrB1, and MfpA focusing on pentapeptide repeats, faces and coils [55, 56]. Alignment of AQnr proteins with other Qnr proteins based on pentapeptide repeats, faces and coils from the structures, revealed the conservation of DNA-mimicking structures in all AQnr families (Fig. 4A). Nine coils constituted by four faces were conserved in all AQnr proteins, with each face exhibiting pentapeptide repeat sequences. Intrachain extension loops 1 and 2 were identified in all AQnr proteins, similar to AhQnr, but not observed in MfpA [32] (Fig. 4A). Overall structure comparison demonstrated that structures and electrostatic surface representation of AQnr proteins were nearly identical to those of AhQnr and QnrB1 (Fig. 4B). These results suggest that novel AQnr proteins (AQnrBE1, AQnrBE3, AQnrBE4, and AQnrBE5) could function as quinolone resistance determinants as shown in AhQnr and QnrB1 [32, 36].

### Quinolone Resistance Analysis of Novel Qnr Family Proteins

To evaluate the impact of AQnr proteins on quinolone resistance, changes in the resistance were monitored upon heterologous expression of the corresponding *aqnr* genes. AQnrBE2, closely resembling the functionally characterized AhQnr (99.07%), served as a positive control. The gene encoding AQnrBE1 (AQnr-12 family), AQnrBE2 (AhQnr family), AQnrBE3 (AQnr-2 family), AQnrBE4 (AQnr-3 family), or AQnrBE5 (AQnr-9 family) was inserted into a pHSG398 plasmid, known for low-level and constitutive expression in *E. coli* [62]. Eight quinolone antibiotics with varying generations and applications, including nalidixic acid, norfloxacin, ofloxacin, levofloxacin, moxifloxacin, enrofloxacin, ciprofloxacin, and sarafloxacin, were employed to determine MIC. *E. coli* TOP10 and *E. coli* TOP10 carrying the intact plasmid (pHSG398) were used as controls. All AQnr proteins of *Aeromonas* isolates exhibited 2- to 16-fold increased resistance against various quinolone antibiotics compared to the controls. The resistance against moxifloxacin and enrofloxacin increased dramatically (16-fold), while the resistance against nalidixic acid showed the least increase (2-fold) (Table 1). There was no difference between AQnrBE2 (AhQnr) and novel AQnr proteins except for the slight elevated sarafloxacin resistance caused by AQnrBE3. This finding underscores that AQnr families are indeed functional resistance determinants (Table 1). Elevated sarafloxain resistance by AQnrBE3 implies that diverse AQnr proteins may confer extended and enhanced resistance spectra due to sequence variation.

## Discussion

In this study, five *Aeromonas* spp. exhibiting MDR phenotypes were isolated from aquatic environmental sites, and their taxonomic assignment and ARG repertories were investigated based on the complete genome sequences. Genome-based phylogenetic analyses accurately assigned the isolates to five distinct species. Despite relatively modest ARG repertoires, these *Aeromonas* spp. demonstrated MDR phenotypes, suggesting the presence of diverse intrinsic resistance mechanisms in these environmental isolates.

Using relaxed criteria in the RGI analyses, Qnr-like proteins were suggested as potential quinolone resistance determinants of the isolates. Previous studies, including AhQnr, have identified Qnr-like proteins in *A. hydrophila*, *A. salmonicida*, *A. veronii*, and *A, caviae*, which exhibit distinct protein sequences from other Qnr proteins [31, 40]. Expanding upon this knowledge, our comprehensive analysis of all available *Aeromonas* genomes, alongside environmental isolates, sheds light on the diversity and conservation of Qnr proteins within the genus. Through classification based on protein sequences, taxonomic distribution of AQnr proteins and their functional characterization, it becomes evident that AQnr proteins represent conserved and intrinsic quinolone resistance determinants in the genus. It has been suggested that current mobile *qnr* genes may have originated from aquatic bacteria such as *Shewanella* sp. and *Vibro* spp. [37, 63]. *Aeromonas* is one of the most important aquatic bacteria responsible for ARG dissemination in the environment [17, 18], indicating that the thorough understanding and surveillance of these intrinsic determinants are demanded prior to their emergence in pathogenic bacteria. Although the contribution of quinolone resistance by the five AQnr proteins of isolates was evaluated, the conserved structure of all AQnr families suggest that they should collectively be considered quinolone resistance determinants demanding surveillance. Furthermore, the presence of AQnr-6 as a paralog in various species implied the indication of potential mobilization of AQnr families within the genus, not yet into other genera.

The acquisition of PMQR *qnr* genes in some of *Aeromonas* spp., alongside the presence of intrinsic AQnr, raised questions regarding the function of AQnr in this genus. The low quinolone resistance observed in the isolates suggests that AQnr expression may be insufficient to confer resistance in these strains. AQnr proteins are assumed as a silent resistance determinant, and further investigations into their expression and regulation will provide their inherent roles in the genus. Additionally, the conserved nature of Qnr proteins across *Aeromonas* species, highlight their potentials as reliable targets for studying the cellular function of pentapeptide repeat proteins [32, 36, 64].

In summary, thorough comparative genome analyses and functional characterization, novel Qnr proteins in environmental *Aeromonas* spp., were characterized, revealing the conservation and intrinsic nature of AQnr proteins as quinolone resistance determinants in the genus *Aeromonas*. Surveillance of diverse AQnr families is essential for understanding and mitigating the spread of antibiotic resistance from this genus.

## Supplemental Materials

Supplementary data for this paper are available on-line only at http://jmb.or.kr.



## Figures and Tables

**Table 1:** ARG repertoires in environmental *Aeromonas* spp. Isolates.

Strain	Target antibiotic*					
	Glycopeptide antibiotic	Peptide antibiotic	Phosphonic acid antibiotic	Tetracycline antibiotic	β-Lactam antibiotic	Multidrug
BE1	*vanT*	-	-	-	*bla*_TRU-1_	*adeF*, *rsmA*
BE2	*vanT*	*arnT*	-	-	*bla*_cepS_, *bla*_OXA-726_	*adeF*, *rsmA*
BE3	*vanT*	-	*fosA8*	*tet(E)*	*bla*_MOX-12_, *bla*_OXA-504_	*adeF*, *rsmA*
BE4	*vanT*	*arnT*	-	-	*bla*_TRU-1_, *cphA5*	*adeF*, *rsmA*
BE5	*vanT*	-	-	-	*bla*_TRU-1_	*adeF*, *rsmA*

*Classification based on the Comprehensive Antibiotic Resistance Database (https://card.mcmaster.ca/)

**Table 2:** The contribution of AQnr proteins from environmental *Aeromonas* spp. isolates to quinolone resistance.

Strains	Minimum inhibitory concentrations (μg/ml)*							
	Nalidixic acid	Norfloxacin	Ofloxacin	Levofloxacin	Moxifloxacin	Enrofloxacin	Ciprofloxacin	Sarafloxacin
*E. coli* TOP10	2.000	0.031	0.016	0.008	0.008	0.004	0.004	0.008
*E. coli* TOP10 pHSG398	2.000	0.031	0.016	0.008	0.008	0.004	0.004	0.008
*E. coli* TOP10 pHSG398-aqnrBE1	4.000 (2-fold)	0.125 (4-fold)	0.063 (4-fold)	0.031 (4-fold)	0.125 (16-fold)	0.063 (16-fold)	0.016 (4-fold)	0.031 (4-fold)
*E. coli* TOP10 pHSG398-aqnrBE2	4.000 (2-fold)	0.125 (4-fold)	0.063 (4-fold)	0.031 (4-fold)	0.125 (16-fold)	0.063 (16-fold)	0.016 (4-fold)	0.031 (4-fold)
*E. coli* TOP10 pHSG398-aqnrBE3	4.000 (2-fold)	0.125 (4-fold)	0.063 (4-fold)	0.031 (4-fold)	0.125 (16-fold)	0.063 (16-fold)	0.016 (4-fold)	0.063 (8-fold)
*E. coli* TOP10 pHSG398-aqnrBE4	4.000 (2-fold)	0.125 (4-fold)	0.063 (4-fold)	0.031 (4-fold)	0.125 (16-fold)	0.063 (16-fold)	0.016 (4-fold)	0.031 (4-fold)
*E. coli* TOP10 pHSG398-aqnrBE5	4.000 (2-fold)	0.125 (4-fold)	0.063 (4-fold)	0.031 (4-fold)	0.125 (16-fold)	0.063 (16-fold)	0.016 (4-fold)	0.031 (4-fold)

*Obtained using the agar dilution method
